# Pseudogene RPL32P3 regulates the blood–tumor barrier permeability via the YBX2/HNF4G axis

**DOI:** 10.1038/s41420-021-00758-9

**Published:** 2021-11-24

**Authors:** Ye Ding, Xiaobai Liu, Chunqing Yang, Xuelei Ruan, Di Wang, Yunhui Liu, Xiuli Shang, Qianshuo Liu, Shuyuan Shen, Lu Zhu, Yixue Xue

**Affiliations:** 1 Department of Neurobiology, School of life Sciences, China Medical University, Shenyang, 110122 China; 2 Key Laboratory of Cell Biology, Ministry of Public Health of China, China Medical University, Shenyang, 110122 China; 3 Key Laboratory of Medical Cell Biology, Ministry of Education of China, China Medical University, Shenyang, 110122 China; 4 Department of Neurosurgery, Shengjing Hospital of China Medical University, Shenyang, 110004 China; 5 Key Laboratory of Neuro-oncology in Liaoning Province, Shenyang, 110004 China; 6 Department of Neurology, The First Affiliated Hospital of China Medical University, Shenyang, 110001 China

**Keywords:** CNS cancer, Blood-brain barrier

## Abstract

The existence of the blood–tumor barrier (BTB) severely hinders the transport of anti-tumor drugs to brain tumor tissues. Selectively opening BTB is of great significance to improve the chemotherapy effect of glioma. Pseudogenes have been recognized as important regulators in various biologic processes. In this study, we identified that ribosomal protein L32 pseudogene 3 (RPL32P3) was highly expressed in glioma-exposed endothelial cells (GECs). Knockdown of RPL32P3 decreased the expression of tight junction-related proteins (TJPs) and increased BTB permeability. Subsequent analysis of the underlying mechanism indicated that RPL32P3 recruited lysine methyltransferase 2 A (KMT2A) to the Y-box binding protein 2 (YBX2) promoter region and mediated H3K4me3 to promote YBX2 transcription. Highly expressed YBX2 bound and stabilized hepatocyte nuclear factor 4 gamma (HNF4G) mRNA. Highly expressed HNF4G directly bound to the promoters of TJPs ZO-1, occludin and claudin-5 to promote their transcriptional activities and regulated BTB permeability. The simultaneous knockdown of RPL32P3, YBX2, and HNF4G combined with doxorubicin (DOX) increased the apoptosis of glioma cells. In conclusion, the current study indicated that RPL32P3 knockdown increased BTB permeability through the YBX2/HNF4G pathway. These findings may provide new targets for the comprehensive treatment of glioma.

## Introduction

Glioma is one of the most common primary malignant tumors in the central nervous system [1, 2]. Chemotherapy plays a crucial role in its treatment [3]. However, due to the existence of blood–tumor barrier (BTB), the transport of drugs to tumor tissue is seriously limited, which attenuates the chemotherapeutic effect [4]. BTB is composed of brain tumor cells, highly specific brain microvascular endothelial cells (ECs) called glioma-exposed endothelial cells (GECs), astrocytes and pericytes, and has some characteristics of blood-brain barrier (BBB). How to open BTB effectively and selectively is of great significance to improve the chemotherapeutic effect of glioma.

Noncoding RNAs (ncRNAs) are composed of a diverse range of RNA species, mainly short ncRNAs and long ncRNAs (lncRNAs). According to the genome composition and the relationship with the coding gene, the transcribed pseudogenes are classified as a type of lncRNAs [5]. The concept of pseudogene was first proposed by Jacq et al. in 1977 [6]. With the development of research, pseudogenes have been implicated in many biological activities, including the occurrence and development of a variety of malignant tumors [7–9]. Therefore, it would be of great significance to identify pseudogenes associated with tumors and clarify their mechanism of action. Ribosomal protein L32 pseudogene 3 (RPL32P3) is mapped to human chromosome 3q21.3, and its parental gene is ribosomal protein L32 (RPL32). So far little is known about their expression and function in GECs.

Lysine methyltransferase 2 A (KMT2A), also known as mixed-lineage leukemia 1 (MLL1), encodes a large protein with a SET domain that contains histone 3 lysine 4 (H3K4) methyltransferase activity [10]. KMT2A is associated with several human malignant tumors such as melanoma and leukemia [11–14]. However, limited data are available regarding the expression and function of KMT2A in GECs.

Y-Box Binding Protein 2 (YBX2) belongs to Y-box binding protein, which takes part in various processes including DNA repair, transcription and translation [15, 16]. YBX2 can act as an RNA binding protein (RBP) and it has been demonstrated to be up-regulated in a variety of tumors, including testicular seminoma, ovarian dysgerminomas, and oral squamous cell carcinoma [17, 18]. The expression and function of YBX2 in GECs have not been reported.

Hepatocyte nuclear factor 4 gamma (HNF4G) is one of the isoforms of hepatocyte nuclear factor 4 (HNF4), which belongs to a superfamily of the orphan nuclear receptor. Nuclear receptors are a class of transcription factors. HNF4G plays a carcinogenic role in a variety of tumors, such as prostate cancer, osteosarcoma, and gastric cancer [19–21]. However, the expression and function of HNF4G in GECs remain unclear.

In the present study, we first investigated the endogenous expression of RPL32P3, YBX2, and HNF4G in GECs. Further, we clarified their interaction and the effects on BTB permeability, as well as the underlying mechanism. This study aims at revealing a new mechanism for regulating the permeability of BTB and providing new ideas for the comprehensive treatment of glioma.

## Results

### RPL32P3 was highly expressed in GECs, knockdown of RPL32P3 decreased the expression levels of tight junction-related proteins and increased BTB permeability

**Fig. 1:**
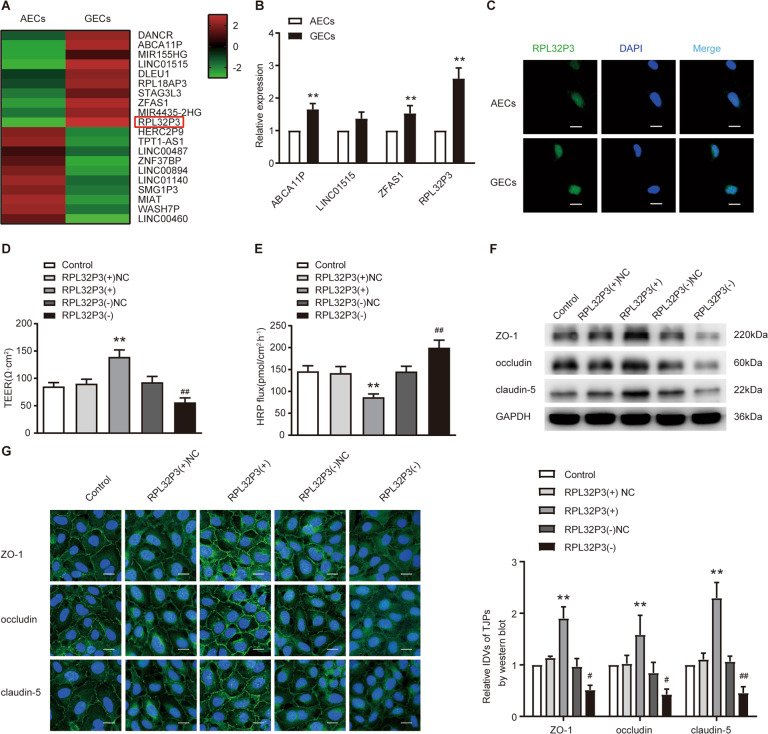
Knockdown of RPL32P3 increased blood–tumor barrier (BTB) permeability in vitro. **A** LncRNAs differentially expressed in astrocyte-exposed ECs (AECs) and glioma-exposed endothelial cells (GECs) were analyzed by lncRNA microarray. Red indicates high expression and green indicates low expression. **B** qRT-PCR was conducted to validate the selected molecules (*n* = 3, each group). **P < 0.01 vs. AECs group. Results were analyzed using the relative quantification (2–ΔΔCT) method. **C** Fluorescence in situ hybridization (FISH) was used to detect the expression and location of RPL32P3 in AECs and GECs (green, RPL32P3; blue, DAPI nuclear staining). Scale bar represents 20 μm. **D**, **E** Effects of RPL32P3 on transendothelial electric resistance (TEER) value and horseradish peroxidase (HRP) flux. Data represent mean ± SD (*n* = 3, each). **P < 0.01 vs. RPL32P3(+)NC group, ^##^*P* < 0.01 vs. RPL32P3 (-)NC group. **F** Effects of RPL32P3 on ZO-1, occludin, and claudin-5 expression levels determined by western blot. Data represent mean ± SD (*n* = 3, each). **P < 0.01 vs. RPL32P3 (+)NC group, ^#^*P* < 0.05 vs. RPL32P3 (-)NC group, ^##^*P* < 0.01 vs. RPL32P3 (-)NC group. Western blot was analyzed in terms of integrated light density values (IDVs). **G** Effects of RPL32P3 on ZO-1, occludin and claudin-5 expression levels and distribution determined by immunofluorescence. Scale bar represents 30 μm.

LncRNA microarray and qRT-PCR were performed and results showed that the expression of RPL32P3 in GECs was significantly higher than that in astrocyte-exposed ECs (AECs) (Fig. 1A, B). Therefore, we selected RPL32P3 for research, whose change in expression was the most obvious. Besides, the expression of its parental gene RPL32 was evaluated in AECs and GECs. No significant difference was detected for RPL32 between AECs and GECs by qRT-PCR (Supplementary Fig. S1A). Fluorescence in situ hybridization (FISH) was performed to investigate not only the expression but also the location of RPL32P3 in AECs and GECs. RPL32P3 was found to be located mainly in nucleus in the cells and was up-regulated in GECs (Fig. 1C). After the nucleus-cytoplasm separation of GECs, results showed that RPL32P3 was mainly located in the nucleus (Supplementary Fig. S1B). To explore the possible function of RPL32P3 in regulating BTB permeability, GECs with stable overexpression or knockdown of RPL32P3 were successfully established. Transfection efficiencies were assessed by qRT-PCR (Supplementary Fig. S2A, B). Subsequently, the transendothelial electric resistance (TEER) value and horseradish peroxidase (HRP) flux were used to test the integrity and permeability of the BTB. The TEER value was significantly decreased whereas HRP flux was significantly increased in the RPL32P3(-) group compared with RPL32P3(-) NC group. And the opposite results were observed in the RPL32P3 (+) group (Fig. 1D, E). Those results indicated that knockdown of RPL32P3 in GECs impaired BTB integrity and increased BTB permeability. Further, western blot analysis showed that the expression of ZO-1, occludin and claudin-5 was significantly decreased in the RPL32P3(-) group compared with RPL32P3(-) NC group in GECs. However, the expression of ZO-1, occludin and claudin-5 increased in the RPL32P3(+) group versus the RPL32P3(+) NC group (Fig. 1F). Similar to the results of western blot, immunofluorescence staining results revealed that compared with the RPL32P3(-) NC group, the expression of ZO-1, occludin and claudin-5 was significantly decreased in the RPL32P3(–) groups. While compared with RPL32P3 (+) NC group, the expression of ZO-1, occludin and claudin-5 in RPL32P3 (+) group was significantly increased (Fig. 1G).

### YBX2 was highly expressed in GECs, knockdown of YBX2 reduced the expression of ZO-1, occludin and claudin-5 in GECs and increased BTB permeability

**Fig. 2:**
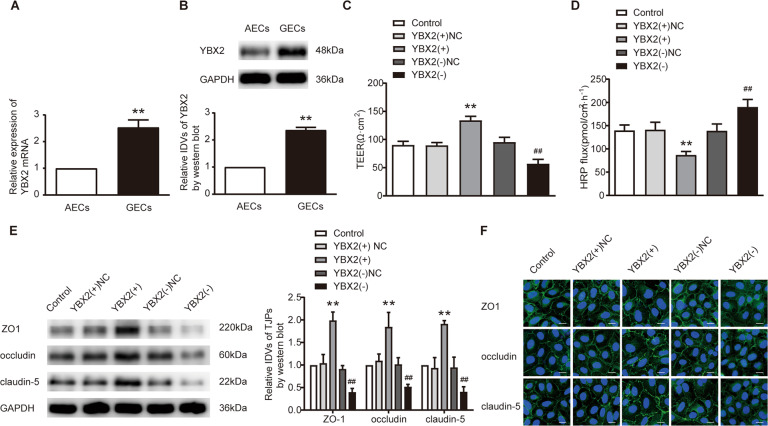
Knockdown of YBX2 increased BTB permeability in vitro. **A, B** The mRNA and protein expression of YBX2 in AECs and GECs were detected by qRT-PCR and western blot. Data represent mean ± SD (*n* = 3, each). **P < 0.01 vs. AECs group. **C**, **D** Effects of YBX2 on TEER value and HRP flux. Data represent mean ± SD (*n* = 3, each). **P < 0.01 vs. YBX2(+)NC group, ^##^*P* < 0.01 vs. YBX2 (-)NC group. **E** Effects of YBX2 on the expression levels of ZO-1, occludin and claudin-5 determined by western blot. Data represent mean ± SD (*n* = 3, each). **P < 0.01 vs. YBX2(+)NC group, ^##^*P* < 0.01 vs. YBX2 (-)NC group. **F** Effects of YBX2 on ZO-1, occludin and claudin-5 expression levels and distribution determined by immunofluorescence. Scale bar represents 30 μm.

With the use of mRNA microarray analysis and qRT-PCR, we verified that YBX2 was significantly decreased in GECs treated with RPL32P3(-) (Supplementary Fig. S3A, B). We further confirmed the decrease of YBX2 protein in RPL32P3(-) group by western blot (Supplementary Fig. S3C). Immunofluorescence and nucleus-cytoplasm separation assays showed that YBX2 was located in both nucleus and cytoplasm and was highly expressed in GECs (Supplementary Fig. S1C, D). YBX2 mRNA and protein both exhibited high expression levels in GECs (Fig. 2A, B). After that, GECs with stable overexpression or knockdown of YBX2 were successfully established. Transfection efficiencies were shown in Supplementary Fig. S2C, D. Knockdown of YBX2 reduced the TEER value and enhanced the HRP flux of GECs, whereas the converse was observed upon YBX2 overexpression (Fig. 2C, D). Moreover, impaired protein levels of ZO-1, occludin and claudin-5 were observed in the YBX2(-) group via western blot (Fig. 2E) and immunofluorescence (Fig. 2F).

### RPL32P3 bound to KMT2A to promote H3K4me3 modification in the YBX2 promoter region

**Fig. 3:**
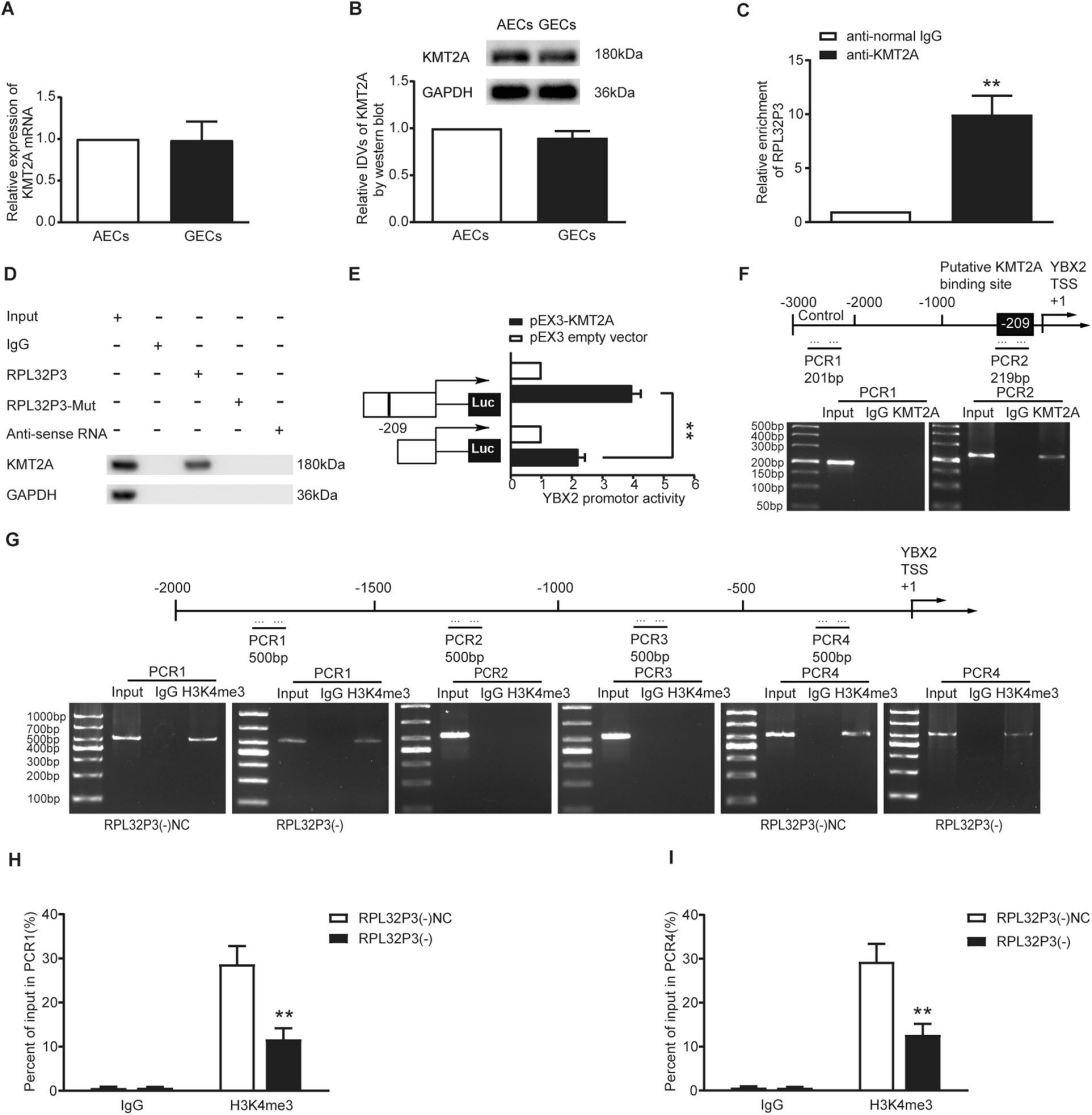
RPL32P3 bound to KMT2A to promote H3K4me3 modification in the YBX2 promoter region. **A**, **B** The mRNA and protein expression of KMT2A in AECs and GECs were detected by qRT-PCR and western blot. Data represent mean ± SD (*n* = 3, each). **C** Enrichment of RPL32P3 in KMT2A immunoprecipitates was measured by RNA immunoprecipitation (RIP) assay. Relative enrichment was measured using qRT-PCR. Data represent means ± SD (*n* = 3, each). **p < 0.01 vs. anti-normal IgG group. **D** RNA pull-down assay confirmed that RPL32P3 bound to KMT2A. KMT2A and GAPDH protein levels in immunoprecipitates with RPL32P3 RNA were evaluated by western blot. **E** Schematic diagram of different reporter plasmids and relative dual-luciferase activities of YBX2. The left side showed the deletion site on YBX2 promoter, and the right side showed the decrease of reporter vector activity after the deletion of the putative binding site. Data represent means ± SD (*n* = 5, each). **P < 0.01. **F** Schematic diagram of YBX2 promoter region from transcription start site (TSS) to 3000 bp upstream in chromatin immunoprecipitation (ChIP) assay. TSS was designated as + 1. Immunoprecipitated DNA was amplified by PCR. Normal rabbit IgG was used as a negative control. **G** YBX2 promoter region was divided into four parts, each part of which was 500 bp on average, from TSS to 2000 bp upstream. H3K4me3 was enriched in PCR1 and PCR4. And reaction bands were detected in RPL32P3(-)NC and RPL32P3(-) groups respectively. **H**, **I** qRT-PCR analysis for the enrichment value of H3K4me3 immunoprecipitated chromatin of PCR1 and PCR4 was calculated. IgG immunoprecipitated chromatin was used as a negative control. Data represent mean ± SD (*n* = 3, each group). **p < 0.01 vs. RPL32P3(-)NC group.

The regulatory effect of RPL32P3 on YBX2 prompted us to explore its internal mechanism. By employing ENCODE (Encyclopedia of DNA Elements) project, we predicted that there were multiple H3K4me3 modification sites on the YBX2 promoter (Supplementary Fig. S4A). And RPISeq predicted that there were interactions between RPL32P3 and KMT2A. Besides, with the use of Animal TFDB, we predicted that H3K4 methyltransferase KMT2A harbored a binding site with the YBX2 promoter (Supplementary Fig. S4B). The results of qRT-PCR and western blot showed that there was no statistical difference in the mRNA and protein expression of KMT2A between AECs and GECs (Fig. 3A, B). Western blot showed that the expression of KMT2A protein had no significant change after RPL32P3 knockdown (Supplementary Fig. S1E). GECs with stable overexpression or knockdown of KMT2A were established. Transfection efficiencies were shown in Supplementary Fig. S2E, F. Western blot showed that the expression of YBX2 protein decreased after KMT2A knockdown (Fig. S1F). As shown by RNA immunoprecipitation (RIP) assay, RPL32P3 was enriched in the anti-KMT2A immunoprecipitate compared with that in the negative control anti-normal IgG group (Fig. 3C). RNA pull-down assay demonstrated that RPL32P3 bound with KMT2A, instead of RPL32P3-mutant and anti-sense RNA (Fig. 3D). Promoter dual-luciferase reporter assay showed that YBX2 promoter activity was remarkably increased after co-transfection with pEX3-KMT2A. And the deletion of the putative binding site with KMT2A (−209 bp) led to a reduction of YBX2 promoter activity (Fig. 3E). Chromatin immunoprecipitation (ChIP) assay showed that there was an interaction of KMT2A with the putative binding site of YBX2 (Fig. 3F). In a further ChIP assay, the YBX2 promoter region was divided into four equal parts from 2000 bp upstream to the transcription start site (TSS), and each part was 500 bp. H3K4me3 antibody and normal rabbit IgG were used to precipitate the protein-DNA complex, then the precipitated DNA was used for PCR. The results showed that H3K4me3 was enriched at −2000 to −1500 bp (PCR1) and −500 bp to TSS (PCR4) of the YBX2 promoter (Fig. 3G). Afterward qRT-PCR was performed to measure the percentage of PCR1 and PCR4 products amplified with the DNA precipitated by H3K4me3 antibody relative to its corresponding input (Fig. 3H, I). The results showed that both PCR1 and PCR4 products decreased significantly after RPL32P3 knockdown.

### YBX2 participated in the process of RPL32P3 regulating BTB permeability

**Fig. 4:**
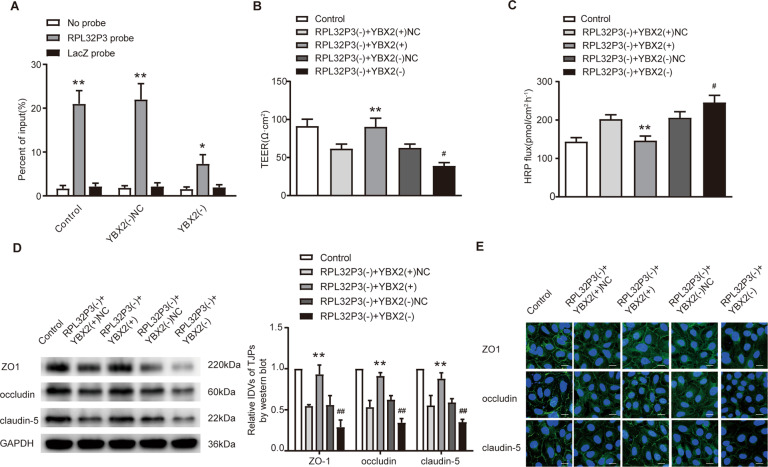
YBX2 participated in the process of RPL32P3 regulating BTB permeability. **A** Chromatin isolation by RNA purification (ChIRP) assays showed that RPL32P3 had a significant occupancy on the YBX2 promoter. The bound DNA was detected by qRT-PCR with specific primers against the YBX2 promoter. Data represent mean ± SD (*n* = 3, each group). *p < 0.05 vs. LacZ probe, **p < 0.01 vs. LacZ probe. **B**, **C** Effects of RPL32P3 and YBX2 on TEER value and HRP flux. **p < 0.01 vs. RPL32P3(-) + YBX2(+)NC group, ^#^*P* < 0.05 vs. RPL32P3(-) + YBX2 (-)NC group. **D** Effects of RPL32P3 and YBX2 on the expression levels of ZO-1, occludin and claudin-5 determined by western blot. Data represent mean ± SD (*n* = 3, each group). **p < 0.01 vs. RPL32P3(-) + YBX2(+)NC group, ^##^*p* < 0.01 vs. RPL32P3(-) + YBX2(-)NC group. **E** Effects of RPL32P3 and YBX2 on ZO-1, occludin and claudin-5 expression levels and distribution determined by immunofluorescence. Scale bar represents 30 μm.

We next performed chromatin isolation by RNA purification (ChIRP) assay to verify the interaction between RPL32P3 and YBX2. The results showed that antisense probe against RPL32P3 bound and effectively captured RPL32P3 (Supplementary Fig. S1G). And RPL32P3 had a significant occupancy on the YBX2 promoter, which decreased obviously after YBX2 knockdown (Fig. 4A). These results indicated that RPL32P3 could physiologically associate with the promoter sequences of YBX2. Finally, a co-transfection assay of RPL32P3 and YBX2 was performed. Compared with knockdown of RPL32P3 alone, knockdown of RPL32P3 combined with knockdown of YBX2 further reduced TEER value and the expression of TJPs, and increased HRP flux. While overexpression of YBX2 could reverse the changes of TEER value, HRP flux and TJPs expression induced by knockdown of RPL32P3 (Fig. 4B-E). No significant differences were found in the effects of the control groups of RPL32P3 and YBX2 on the expression levels of TJPs (Supplementary Fig. S5A).

### HNF4G was up-regulated in GECs, and knockdown of HNF4G increased the BTB permeability

**Fig. 5:**
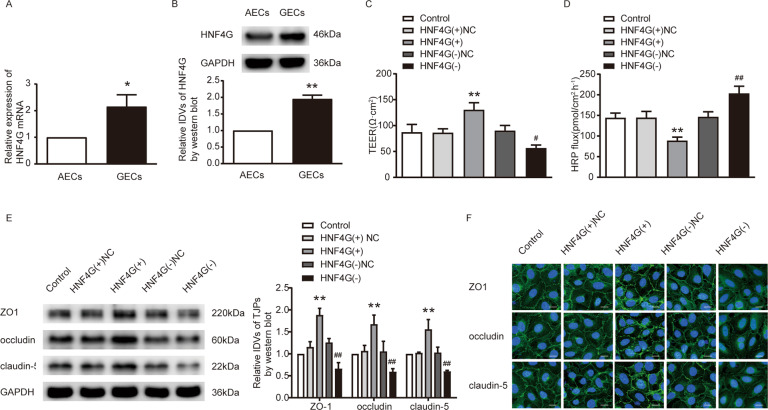
Knockdown of HNF4G increased BTB permeability in vitro. **A**, **B** The mRNA and protein expression levels of HNF4G were up-regulated in GECs than in AECs by qRT-PCR and western blot. Data represent mean ± SD (*n* = 3, each). *P < 0.05 vs. AECs group, **P < 0.01 vs. AECs group. **C**, **D** Effects of HNF4G on TEER value and HRP flux. Data represent mean ± SD (*n* = 3, each). **P < 0.01 vs. HNF4G(+)NC group, ^#^*P* < 0.05 vs. HNF4G(-)NC group, ^##^*P* < 0.01 vs. HNF4G(-)NC group. **E** Effects of HNF4G on the expression levels of ZO-1, occludin and claudin-5 determined by western blot. Data represent mean ± SD (*n* = 3, each). **P < 0.01 vs. HNF4G(+)NC group, ^##^*P* < 0.01 vs. HNF4G(-)NC group. **F** Effects of HNF4G on ZO-1, occludin and claudin-5 expression levels and distribution determined by immunofluorescence. Scale bar represents 30 μm.

With the use of mRNA microarray analysis, qRT-PCR and western blot, HNF4G was selected for research (Supplementary Fig. S3D-F). Immunofluorescence and nucleus-cytoplasm separation assays showed that HNF4G was located in both nucleus and cytoplasm and was up-regulated in GECs (Supplementary Fig. S1H, I). HNF4G mRNA and protein were highly expressed in GECs compared with those in AECs (Fig. 5A, B). GECs with HNF4G stable overexpression or knockdown were successfully established. Transfection efficiencies were shown in Supplementary Fig. S2G, H. HNF4G knockdown reduced the TEER value and enhanced the HRP flux. The HNF4G(+) group showed the opposite results (Fig. 5C, D). The results of qRT-PCR and Western blot showed that the expression of ZO-1, occludin and claudin-5 in the HNF4G(-) group decreased, while the HNF4G(+) group showed the opposite results (Supplementary Fig. S1J and Fig. 5E). Immunofluorescence staining showed similar results (Fig. 5F).

### YBX2 targeted and stabilized HNF4G, HNF4G was involved in the regulation of BTB permeability by YBX2

**Fig. 6:**
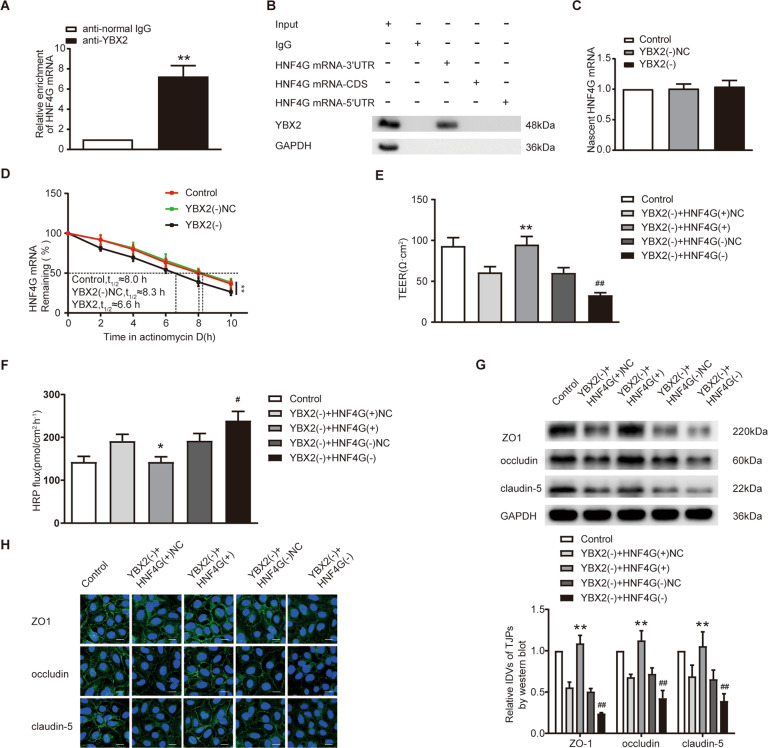
Knockdown of YBX2 decreased HNF4G stability, and HNF4G participated in the process of YBX2 regulating BTB permeability. **A** Enrichment of HNF4G mRNA in anti-YBX2 immunoprecipitates was measured by RIP assay. Relative enrichment of HNF4G mRNA was measured using qRT-PCR. Data represent mean ± SD (*n* = 3, each). **P < 0.01 vs. anti-normal IgG group. **B** RNA pull-down assay confirmed that YBX2 bound to HNF4G mRNA. YBX2 and GAPDH protein levels in immunoprecipitates with HNF4G mRNA were evaluated by western blot. **C** Nascent HNF4G mRNA was labeled and captured by nascent RNA capture assay. No significant difference of HNF4G mRNA was found in YBX2 knockdown GECs. **D** Knockdown of YBX2 decreased the half-life of HNF4G mRNA. Data represent mean ± SD (*n* = 3, each). **P < 0.01 vs. YBX2(-)NC group. **E**, **F** Effects of YBX2 and HNF4G on TEER value and HRP flux. *p < 0.05 vs. YBX2(-) + HNF4G(+)NC group, **p < 0.01 vs. YBX2(-) + HNF4G(+)NC group, ^#^*p* < 0.05 vs. YBX2(-) + HNF4G(-)NC group, ^##^*p* < 0.01 vs. YBX2(-) + HNF4G(-)NC group. **G** Effects of YBX2 and HNF4G on the expression levels of ZO-1, occludin and claudin-5 determined by western blot. Data represent mean ± SD (*n* = 3, each group). **p < 0.01 vs. YBX2(-) + HNF4G(+)NC group, ^##^*p* < 0.01 vs. YBX2(-) + HNF4G(-)NC group. **H** Effects of YBX2 and HNF4G on ZO-1, occludin and claudin-5 expression levels and distribution determined by immunofluorescence. Scale bar represents 30 μm.

RIP assay indicated that HNF4G mRNA was enriched in the anti-YBX2 group (Fig. 6A). Western blot was performed using the retrieved proteins following RNA pull-down assay, and the result verified the binding of YBX2 and HNF4G mRNA 3’UTR, instead of 5’UTR and coding sequences (CDS) (Fig. 6B). Nascent RNA capture assay showed that the effect of YBX2 knockdown on newly synthesized HNF4G mRNA was not statistically significant (Fig. 6C). RNA stability measurement showed that the half-life of HNF4G mRNA was shortened from 8.3 h to 6.6 h after YBX2 knockdown (Fig. 6D). Further co-transfection assay of YBX2 and HNF4G showed that compared with YBX2 knockdown alone, the combination of YBX2 knockdown and HNF4G knockdown further reduced TEER value, increased HRP flux, and further reduced the expression of TJPs. While overexpression of HNF4G could reverse the changes of TEER value, HRP flux and TJPs expression induced by YBX2 knockdown (Fig. 6E-H). No significant differences were observed in the effects of the control groups of YBX2 and HNF4G on the expression levels of TJPs (Supplementary Fig. S5B).

### HNF4G directly bound to the promoters of ZO-1, occludin and claudin-5 to promote their transcription and regulate BTB permeability

**Fig. 7:**
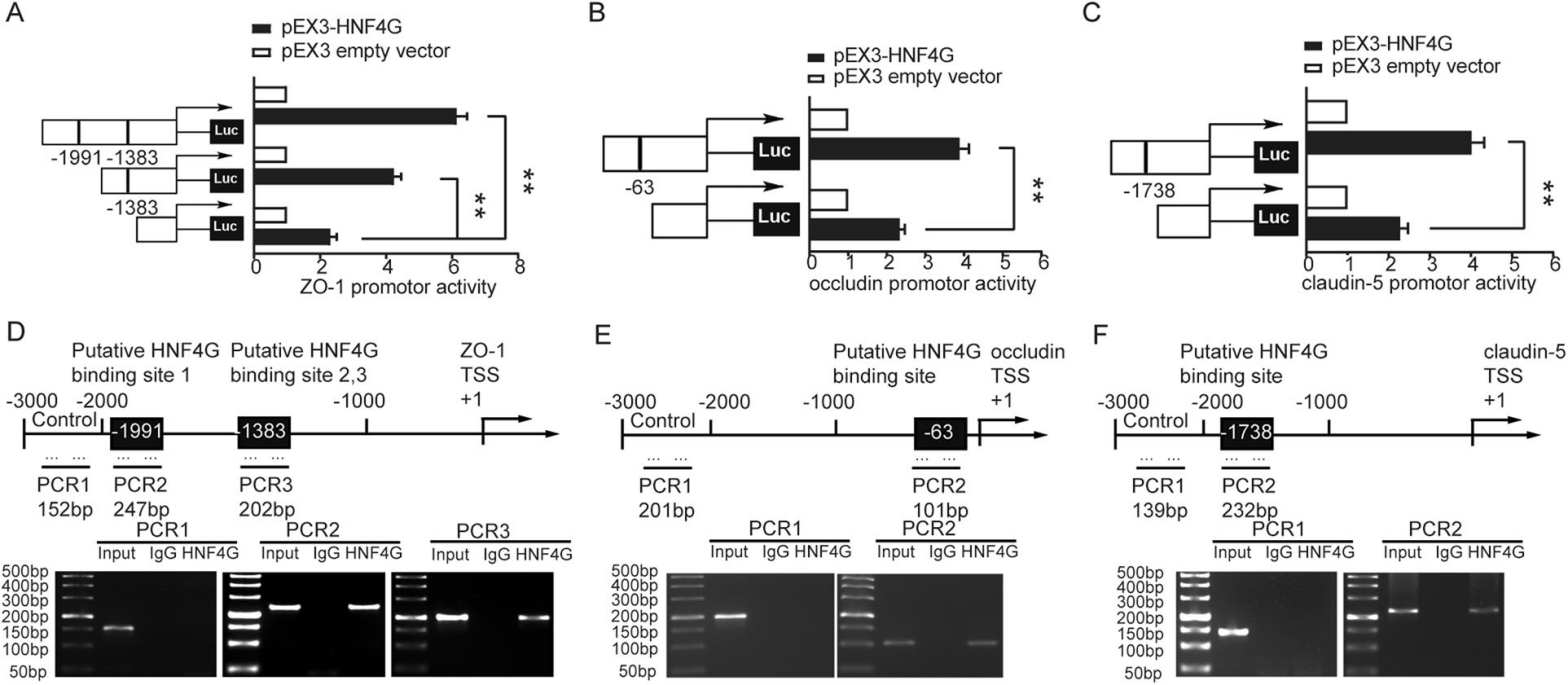
HNF4G bound to ZO-1, occludin and claudin-5 promoters, and inhibited their expression at the transcriptional level. **A**–**C** Schematic diagrams of different reporter plasmids and relative dual-luciferase activities of ZO-1, occludin and claudin-5. The left side showed the deletion sites on the promoters of ZO-1, occludin and claudin-5, and the right side showed the decreases of reporter vector activities after the deletions of the putative binding sites of HNF4G. Data represent means ± SD (*n* = 5, each). **P < 0.01. **D**–**F** Schematic representations of ZO-1, occludin and claudin-5 promoter region from TSS to upstream 3000 bp in ChIP assays. TSS was designated as +1. Immunoprecipitated DNA was amplified by PCR. Normal rabbit IgG was used as a negative control.

HNF4G knockdown reduced the expression of TJPs at mRNA and protein levels, which led us to speculate that HNF4G may directly regulate the expression of TJPs at the transcriptional level. The potential binding sites of TJPs promoters to which HNF4G bound were analyzed with the help of the JASPAR database (Supplementary Fig. S4C-E). As the results of dual-luciferase reporter assays presented, compared with the pEX3 empty vector group, the activities of the ZO-1, occludin and claudin-5 promoters in the pEX3-HNF4G group were significantly increased. Then putative HNF4G binding sites within the ZO-1, occludin and claudin-5 promoters were deleted successively. Deletions of the -1991 bp and the -1383 bp sites resulted in obviously decreased activities of ZO-1 promoter, respectively (Fig. 7A). Deletion of -63 bp site resulted in a significant decrease in the activity of occludin promoter (Fig. 7B). Similarly, the deletion of -1738 bp site significantly reduced the activity of claudin-5 promoter (Fig. 7C). ChIP assays show that HNF4G bound with the ZO-1, occludin and claudin-5 promoters. (Fig. 7D-F).

### Knockdown of RPL32P3, YBX2, and HNF4G alone or in combination promoted doxorubicin-induced glioma cell apoptosis

**Fig. 8:**
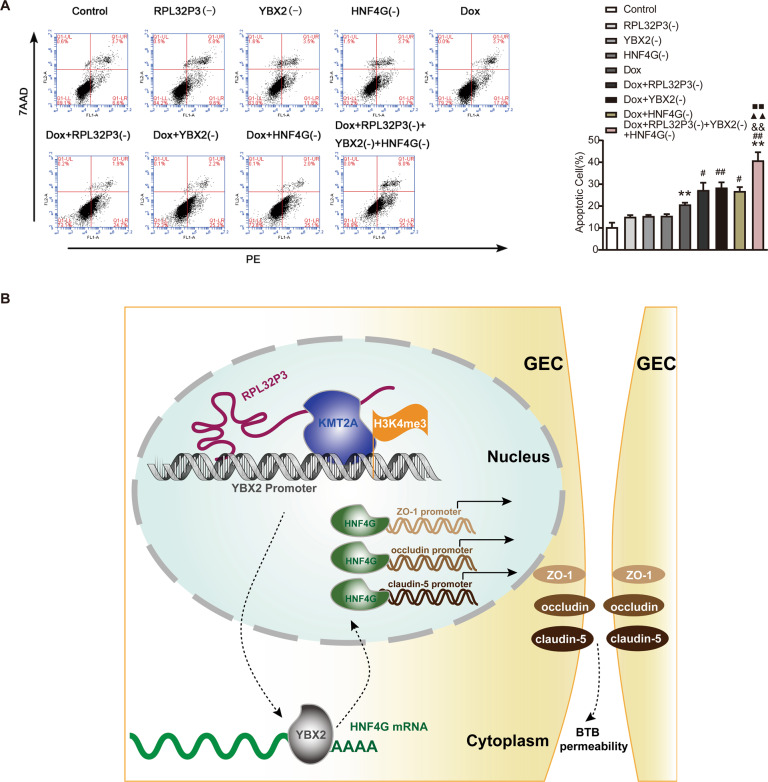
Combined knockdown of RPL32P3, YBX2, and HNF4G increased the effect of doxorubicin (DOX) in promoting apoptosis of U251 cells. **A** Single or combined use of RPL32P3 knockdown, YBX2 knockdown and HNF4G knockdown increased the promoting effect of DOX on the apoptosis rate of U251 cells. Data represent mean ± SD (*n* = 3, each). **P < 0.01 vs. Control group, ^#^*P* < 0.05 vs. DOX group, ^##^*P* < 0.01 vs. DOX group, ^&&^*P* < 0.01 vs. DOX + RPL32P3(-) group, ^▲▲^*P* < 0.01 vs. DOX + YBX2(-) group, ^■■^*P* < 0.01 vs. DOX + HNF4G(-) group. **B** The schematic diagram about the regulating process of RPL32P3/YBX2/HNF4G axis on BTB permeability.

We further studied the effect of different treatment conditions on the apoptosis rate of U251 glioma cells. As shown in Fig. 8A, compared with the control group, there was no significant difference in apoptosis rate among RPL32P3(-) group, YBX2(-) group, and HNF4G(-) group. Compared with the doxorubicin (DOX) group, the respective knockdown of RPL32P3, YBX2, and HNF4G all promoted U251 cells apoptosis caused by DOX. Notably, the combination of RPL32P3 knockdown, YBX2 knockdown, and HNF4G knockdown showed the greatest effect. The schematic diagram of the regulation of RPL32P3/YBX2/HNF4G axis in BTB permeability was shown in Fig. 8B.

## Discussion

In recent years, benefiting from the development of bioinformatics and sequencing technologies, tens of thousands of pseudogenes have been identified. Some pseudogenes are involved in regulating the occurrence and development of glioma. For instance, DUXAP8 is up-regulated in glioma cells, and knockdown of DUXAP8 suppresses the proliferation of glioma [22]. ANXA2P2 is up-regulated in glioma cells, and it modulates the aerobic glycolysis and proliferation of glioma cells through ANXA2P2/miR-9/LDHA axis [23]. This study found for the first time that RPL32P3 was highly expressed in GECs. Knockdown of RPL32P3 decreased ZO-1, occludin and claudin-5 expression and increased BTB permeability. Recent studies have shown that pseudogenes can be distributed and expressed in the nucleus and cytoplasm, and regulate a variety of physiological and pathological processes. Pseudogene RPSAP52 positively regulates the transcription of HMGA2 in the nucleus and acts as a translational co-regulator with LIN28B and HMGA2 mRNAs in the cytoplasm to regulate the function of IGF2BP2 protein; RPSAP52 regulates the HMGA2/IGF2BP2/LIN28B axis through a double mechanism to show the oncogene characteristics in breast cancer, rhabdomyosarcoma, Ewing’s sarcoma and other human tumors [24]. Pseudogene PTENP1 regulates the tumor suppressor PTEN as a ceRNA in the cytoplasm [25]. This study demonstrated for the first time that RPL32P3 was mainly located in the nucleus in human cerebral microvascular ECs. In addition, we found that the expression of RPL32P3 in GECs was higher than that in AECs, but there was no significant difference in the expression of RPL32 between AECs and GECs. This suggested that pseudogene RPL32P3 may regulate BTB permeability in a way independent of its parental gene RPL32.

YBX2 is involved in regulating the biological processes of a variety of tumors. YBX2 is remarkably up-regulated in oral squamous cell carcinoma and is a key target gene for the HOXA11-AS/miR-98-5p axis to regulate oral squamous cell carcinoma progression [26]. YBX2 appears to contribute to molecular subtype-specific splicing in breast cancer [27]. This study found for the first time that YBX2 was highly expressed in GECs. Knockdown of YBX2 decreased the expression of TJPs and TEER value but raised the HRP flux and BTB permeability.

Pseudogenes can perform functions similar to lncRNAs and regulate downstream genes through epigenetic modifications such as histone methylation. Pseudogene DUXAP8 recruits histone methyltransferase EZH2 to the EGR1 promoter region and mediates H3K27me3 to inhibit EGR1 transcription. Meanwhile, it recruits histone demethylase LSD1 to the RHOB promoter region and mediates H3K42 demethylation to silence RHOB, thus promoting non-small-cell lung cancer cell proliferation and invasion [28]. In this study, RIP and RNA pull-down assays proved the binding of RPL32P3 to the H3K4 methyltransferase KMT2A. ChIP assay proved the binding of KMT2A to the YBX2 promoter and the occurrence of the H3K4me3 modification on the YBX2 promoter. The result of further ChIP assay revealed that H3K4me3 enrichment mainly occurred from 2000 to 1500 bp upstream and from 500 bp upstream to TSS of YBX2 promoter. RPL32P3 knockdown reduced H3K4me3 enrichment and thus YBX2 expression. H3K4me3 is one of the most extensively studied histone modifications and is a hallmark of the promoters of actively transcribing and poised genes [29]. Mounting evidence revealed an intimate relationship between H3K4me3 and a class of vertebrate-specific gene regulatory elements called CpG islands. H3K4me3 is present at most of CpG islands, and the targeting and deposition of H3K4me3 in vertebrates are inherently linked to CpG islands. CpG islands shape the pattern of H3K4me3 after its deposition, and H3K4me3 can affect the chromatin architecture around CpG island-associated gene promoters to influence gene expression [30]. In this study, based on the results of the bioinformatics software MethPrimer (Supplementary Fig. S4F), the CpG islands on the YBX2 promoter were mainly located upstream 14-576 bp and upstream 1650-1956 bp, which were almost consistent with the results of the H3K4me3 enrichment positions in ChIP assay. Furthermore, ChIRP assay proved that RPL32P3 existed in the promoter region of YBX2. YBX2 knockdown further reduced BTB permeability on the basis of RPL32P3 knockdown, while YBX2 overexpression reversed the decrease of BTB permeability caused by RPL32P3 knockdown. Collectively, these findings supported that RPL32P3 mediated H3K4me3 by binding and recruiting KMT2A to the promoter region of YBX2, thus promoting the transcription of YBX2 and regulating BTB permeability. Similar to our findings, a previous study has shown that lncRNA FEZF1-AS1 recruits histone demethylase LSD1 at the p21 promoter, causing H3K4me2 demethylation and inhibiting p21 transcription, thereby promoting the proliferation of gastric cancer cells [31].

RBPs mainly play a regulatory role at different levels by interacting with different RNAs, including noncoding RNA and mRNA. Based on mRNA microarray analysis and bioinformatics tool RBPmap software, we performed RIP and RNA pull-down assays, proving the binding effect between YBX2 and HNF4G mRNA. YB proteins are involved in stability and decay regulation of mRNA [32]. Ybx2-knockout mice could form brown adipose tissue but failed to express a full thermogenic program, Ybx2 targeted and stabilized Pgc1a mRNA to control brown adipose tissue activation [33]. In this study, we found that YBX2 knockdown did not affect the synthesis of HNF4G mRNA but shortened its half-life, suggesting that YBX2 knockdown decreased the stability of HNF4G mRNA. Co-transfection assays further proved that HNF4G knockdown promoted the decrease of BTB permeability induced by YBX2 knockdown, while HNF4G overexpression reversed the decrease of BTB permeability induced by YBX2 knockdown. Similar to the results of this study, RBP-AGO2 promoted the proliferation of hepatocellular carcinoma cells by binding and stabilizing the transcription factor MYC mRNA [34].

HNF4G plays a role in a variety of physiological and pathological processes, such as regulating chromatin accessibility, tumorigenesis and development [35, 36]. In this study, we found that HNF4G was highly expressed in GECs. Knockdown of HNF4G decreased ZO-1, occludin and claudin-5 expression and increased BTB permeability. Studies have shown that the HNF4 family is related to the regulation of cell barrier or permeability. In pancreatic ductal adenocarcinoma, the role of HNF4G overexpression in promoting metastasis may be through regulating the cell-cell junction pathway [37]. HNF4α triggered formation of the functional TJPs occludin and claudin-6/7 and the establishment of polarized epithelial morphology in F9 embryonal carcinoma cells [38]. As nuclear receptor transcription factors, HNF4 can directly regulate the transcription of the target gene by combining with the target gene promoter. HNF4α negatively regulated the transcription of CTGF by binding to its promoter during hepatocyte regeneration [39]. HNF4G activated the transcription of HAS2 by interacting with the promoter/enhancer region of HAS2 to promote the growth and invasion of bladder cancer cells [40]. Based on the bioinformatics tool JASPAR database, we found five potential binding sites of HNF4G in the promoter regions of TJPs. The dual-luciferase reporter and ChIP assays confirmed that HNF4G bound to the promoter regions of TJPs and activated their transcriptions.

DOX is a classic anthracycline antibiotic, which has a potent anti-tumor effect on a variety of tumors [41]. The existence of BTB affects the chemotherapy effect of DOX on glioma. In this study, by establishing the BTB model in vitro, we found that the simultaneous knockdown of RPL32P3, YBX2, and HNF4G combined with DOX could significantly increase the apoptosis rate of glioma cells. The results suggested that the combined application of knockdown of RPL32P3, YBX2, and HNF4G could promote the passage of DOX through BTB and enhance the inhibitory effect of DOX on glioma cells.

Taken together, our study demonstrated for the first time that RPL32P3 was highly expressed in GECs. RPL32P3 recruited KMT2A to the YBX2 promoter region and mediated H3K4me3 to promote YBX2 transcription. Highly expressed YBX2 bound and stabilized HNF4G mRNA. Highly expressed HNF4G directly bound to the promoters of ZO-1, occludin and claudin-5 to promote their transcriptional activities and regulate BTB permeability. The combination of simultaneous knockdown of RPL32P3, YBX2, and HNF4G with DOX significantly induced U251 glioma cells apoptosis and produced the strongest effects.

## Materials and methods

### Cell culture

The immortalized human cerebral microvascular endothelial cell line hCMEC/D3 was friendly provided by Dr. Couraud (Institut Cochin, Paris, France). Human glioma cell line U251 and human embryonic kidney 293 T (HEK293T) cell line were purchased from the Shanghai Institutes for Biological Sciences Cell Resource Center. Normal human astrocytes cell line (NHAs) was purchased from ScienCell Research Laboratories (Carlsbad, CA, USA). All cells were cultured at 37 °C in a humidified incubator of 5% CO2 and refreshed medium every two days. For details, see Supplemental Materials and Methods.

### Establishment of in vitro BTB and BBB model

In vitro BTB models were established by co-culture of ECs and U251 cells, and BBB models were established by co-culture of ECs and NHAs as described previously [42]. The glioma cells co-cultured ECs (glioma-exposed ECs) were called GECs. The NHAs co-cultured ECs (astrocyte-exposed ECs) were called AECs. For details, see Supplemental Materials and Methods.

### Quantitative real-time PCR

All quantitative real-time PCR (qRT-PCR) reactions were performed as previously described [42]. For details, see Supplemental Materials and Methods. Sequences of primers were shown in Supplementary Table S1.

### Western blot assay

Western blot assay was performed as previously described [42]. For details, see Supplemental Materials and Methods.

### Immunofluorescence assay

Immunofluorescence assay was performed as previously described [42]. See Supplemental Materials and Methods for details.

### Fluorescence in situ hybridization (FISH)

RPL32P3 probe (green-labeled; GenePharma, Shanghai, China) was used. FISH was performed as previously described [43]. For details, see Supplemental Materials and Methods.

### Nucleus-cytoplasm separation assay

Nucleus-cytoplasm separation assay was performed as previously described [43]. The RNA and protein in the nucleus and cytoplasm of GECs were extracted. RPL32P3, YBX2 mRNA, and HNF4G mRNA were detected by qRT-PCR. YBX2 and HNF4G proteins were detected by western blot. For details, see Supplemental Materials and Methods.

### Transendothelial electric resistance (TEER) assay

After in vitro BTB models were established, TEER assays were performed as previously described [42]. For details, see Supplemental Materials and Methods.

### Horseradish peroxidase (HRP) flux assay

HRP flux assay was performed as previously described [42]. For details, see Supplemental Materials and Methods.

### Cell transfection

Cell transfection was performed as previously described [42]. For details, see Supplemental Materials and Methods. Sequences of shRNA templates were shown in Supplementary Table S2.

### Human lncRNA and mRNA microarray analysis

Microarray analysis, sample preparation and microarray hybridization were operated by Aksomics Biotech (Shanghai, China).

### Reporter vector construction and dual-luciferase reporter assays

Luciferase assays were performed as previously described [42]. For details, see Supplemental Materials and Methods.

### RNA Immunoprecipitation (RIP) assay

RIP assay was performed as previously described [44]. For details, see Supplemental Materials and Methods.

### RNA pull-down assay

RNA pull-down assay was performed as previously described [44]. For details, see Supplemental Materials and Methods.

### Nascent RNA capture

The nascent RNA capture assay was performed as previously described [43]. For details, see Supplemental Materials and Methods.

### RNA stability measurement

Cells were cultured in the medium containing 2 µg/mL actinomycin D (Act D; APExBIO, Houston, TX, USA) to block the de novo RNA synthesis. Total RNA was extracted from the cells collected at 2, 4, 6, 8, 10 h. The expression of mRNA was detected by qRT-PCR. The half-life of mRNA was detected by its expressing level at a certain time point compared with time 0 h.

### Chromatin immunoprecipitation (ChIP) and ChIP-qPCR

ChIP and ChIP-qPCR assays were performed as previously described [43]. See Supplemental Materials and Methods for details. Sequences of primers were shown in Supplementary Table S3.

### Chromatin isolation by RNA purification (ChIRP)

ChIRP assay was performed as previously described [45]. See Supplemental Materials and Methods for details. Sequences of probes and primers were listed in Supplementary Table S4 and S5, respectively.

### Analysis of apoptosis by flow cytometry

Analysis of apoptosis was performed as previously described [44]. See Supplemental Materials and Methods for details.

### Statistical analysis

GraphPad Prism v8.0.1 (GraphPad, La Jolla, CA) software was used for statistical analysis. All data were expressed as mean ± standard deviation (SD). Statistical analysis was performed using Student’s *t*-test and ANOVA. The difference was considered statistically significant when *p* < 0.05.

## Supplementary information

Supplemental Materials and Methods

Supplemental Tables

Supplementary Figure S1

Supplementary Figure S2

Supplementary Figure S3

Supplementary Figure S4

Supplementary Figure S5

Supplementary Figure Legend

cddiscovery-author-contribution-form

## Data Availability

The data used to support the findings of this study have been included in this published article and its additional files.
