# Supplementary material for: Pseudogene RPL32P3 regulates the blood–tumor barrier permeability via the YBX2/HNF4G axis
Source: Cell Death Discov. 2021 Nov 24;7:367. doi: 10.1038/s41420-021-00758-9 (PMC8613260; doi:10.1038/s41420-021-00758-9)
Supplement: Supplementary file 1 — Supplemental Materials and Methods [file 41420_2021_758_MOESM1_ESM.docx]

**Supplemental Materials and Methods**

**Cell Culture**

ECs were cultured in endothelial basal medium (EBM-2) (Lonza, Walkersville, MD, USA), containing 5% fetal bovine serum (FBS) “Gold” (PAA Laboratories, Pasching, Austria), 1% penicillin-streptomycin (Life Technologies, Paisley, UK), 1% chemically defined lipid concentrate (Life Technologies, Paisley, UK), 1.4μmol/L hydrocortisone (Sigma-Aldrich, St Louis, MO, USA), 5μg/mL ascorbic acid (Sigma-Aldrich, St Louis, MO, USA), 10 mmol/L HEPES (PAA Laboratories, Pasching, Austria) and 1ng/mL human basic fibroblast growth factor (BFGF) (Sigma-Aldrich, St Louis, MO, USA). U251 glioblastoma cells and HEK-293T cells were cultured in Dulbecco's Modified Eagle Medium (DMEM) /high glucose with 10% FBS (Gibco, Carlsbad, CA, USA). NHAs were cultured in astrocyte medium with 10% FBS (Gibco, Carlsbad, CA, USA).

**Establishment of In Vitro BTB and BBB Model**

For the BTB model, U251 cells were seeded onto the six-well culture plate at a density of 2×10^4^ per well for 2 days. Then, ECs were seeded onto the Transwell insert (0.4μm pore size; Corning, Lowell, MA, USA) pretreated with 150µg/mL Cultrex Rat Collagen I (R&D Systems, Minneapolis, MN, USA) at a density of 2 ×10^5^ per well. After that, the inserts were placed in the well of the six-well plates containing U251 cells and co-cultured in EBM-2 medium for 4 days, with the medium being changed every 2 days. For the BBB model, NHAs were seeded onto the six-well culture plate at a density of 2×10^4^ per well for 2 days. And the subsequent process is the same as the BTB model in vitro.

**qRT-PCR**

Total RNAs were separated from AECs and GECs using Trizol reagent (Life Technologies, Carlsbad, CA, USA). For measuring the levels of RPL32P3 (NR_003111.2), RPL32 (NM_000994.4), KMT2A (NM_001197104.2), YBX2 (NM_015982.4) and HNF4G (NM_001330561.2), One-Step SYBR PrimeScript RT-PCR Kit (Perfect Real Time, RR066A; Takara Bio, Kusatsu, Japan) was used. GAPDH was used as an endogenous control. RNA concentration and quality were determined using a Nanodrop Spectrophotometer (ND-100; Thermo Fisher Scientific, Waltham, MA, USA). All qRT-PCR reactions were conducted by the ABI 7500 Fast RT-PCR System (Applied Biosystems, Foster City, CA, USA). Relative expression values were calculated using the relative quantification (2^-ΔΔCt^) method.

**Western Blot Assay**

Total proteins of cells were extracted with RIPA buffer (Beyotime Institute of Biotechnology, Jiangsu, China) supplemented with protease inhibitors (10mg/mL aprotinin, 10mg/mL PMSF, and 50 mM sodium orthovanadate) and centrifuged at 17,000×g for 30 min at 4℃. After that, the BCA protein assay kit (Beyotime Institute of Biotechnology, Jiangsu, China) was used to determine the protein concentration of the supernatant. The proteins were fractionated using SDS-PAGE electrophoresis and transferred onto polyvinylidene fluoride (PVDF) membrane (Millipore, Shanghai, China). After blocking with 5% nonfat dry milk in Tris-buffered saline/Tween 20 (TBST) for 2 h, membranes were incubated with primary antibodies as follows: KMT2A (1:1,000; 14197, Cell Signaling Technology), YBX2 (1:800; 13538-1-AP, Proteintech), HNF4G (1:800; 25801-1-AP, Proteintech), GAPDH (1:20,000; 60004-1-Ig, Proteintech), H3 (1:500; AF0863, Affinity), ZO-1(1:300; 61-7300, Life Technologies), occludin (1:1,000; 27260-1-AP， Proteintech), and claudin-5 (1:300; 35-2500, Life Technologies) at 4℃ overnight. The membranes were washed for three times with TBST and then respectively incubated with appropriate HRP-conjugated secondary antibody at room temperature for 2 h as follows: goat anti-rabbit (1:10,000; SA00001-2, Proteintech) or goat anti-mouse (1:10,000; SA00001-1, Proteintech). After washing for three times with TBST, these protein blots were visualized by an enhanced chemiluminescence (ECL) kit (Santa Cruz Biotechnology, Dallas, TX) and detected by ECL Detection Systems (Thermo Scientific, Beijing, China). Then, the protein bands were scanned using ChemiImager 5500 V2.03 software, and integrated light density values (IDVs) were calculated by FluorChem 2.0 software and normalized with those of GAPDH.

**Immunofluorescence Assay**

Cells were fixed with 4% paraformaldehyde for 30 min at room temperature and permeated in PBS containing 0.2% Triton X-100 for 5 min, followed by incubation in 5% BSA blocking buffer for 2 h at room temperature. Then, cells were incubated with primary antibodies against ZO-1 (1:50; 61-7300, Life Technologies), occludin (1:100; 27260-1-AP，Proteintech) and claudin-5 (1:50; 35-2500, Life Technologies) overnight at 4 °C. After washing with PBS/Tween 20 (PBST) for three times, cells were incubated with Alexa-Fluor-488-labeled goat anti-mouse IgG secondary antibody (1:500; A0428, Beyotime Institute of Biotechnology) or anti-rabbit IgG secondary antibody (1:500; A0423, Beyotime Institute of Biotechnology) for 2 h at room temperature in the dark. Then the nuclei were counterstained with 0.5µg/mL DAPI (Beyotime Institute of Biotechnology, Jiangsu, China) for 5 min. Staining was visualized using confocal microscopy (Nikon, C2).

**FISH**

Cells were seeded onto the confocal dish at a density of 1×10^5^ cells per well and then incubated the cells at 37°C overnight. Discard culture medium and wash twice with PBS for 5 min each time. Add 100 μL of 4% paraformaldehyde to each well, and fix for 15 min at room temperature. Add 100 μL of 0.1% Buffer A (currently used) to each well for 15 min at room temperature. Wash twice with PBS for 5 min each time, add 100 μL of 2×Buffer C to each well, and place in a 37°C incubator for 30 min. Add 100 μL of the probe and buffer E mixture to each well, denature at 73°C for 5 min, and incubate overnight in a 37°C incubator for 12-16 h. Add 100 μL of 0.1% Buffer F to each well for 5 min. Then add 100 μL of 2×/1×Buffer C to each well for 5 min successively. Add 100 μL of diluted DAPI working solution to each well, stain for 20 min in the dark and add 100 μL PBS. Staining was visualized using confocal microscopy (Nikon, C2).

**Nucleus-cytoplasm Separation Assay**

The nucleus and cytoplasm fractions of GECs were separated by using the RNA and protein isolation (PARIS) Kit (Invitrogen, Carlsbad, CA, USA). In brief, GECs were washed three times with PBS and incubated on ice for 5-10 min. Incubated the lysates for another 5 min after the centrifugation at 500×g, and then respectively collected the pellet and supernatant as the nucleus and cytoplasm fractions. The extracted lysates of nucleus and cytoplasm were stored at -80℃ for further study. RNA and proteins were both extracted from the nucleus and cytoplasm fractions. qRT-PCR was used to detect the expression of RPL32P3, YBX2 mRNA and HNF4G mRNA in nucleus and cytoplasm of GECs. U6 and GAPDH were served as nucleus control and cytoplasm control, respectively. Western blot was used to detect the expression of YBX2 and HNF4G protein in nucleus and cytoplasm of GECs, Histone H3 and GAPDH were served as nucleus control and cytoplasm control, respectively.

**TEER Assay**

In order to ensure temperature equilibration and uniformity of the culture environment, TEER was recorded after 30 min at room temperature and the culture medium was refreshed before each measurement. The final resistance (Ω▪cm^2^) was calculated by subtracting background resistance from measured barrier resistance, and multiplied by the effective surface area of the filter membrane.

**HRP Flux Assay**

1 mL of serum-free EBM-2 medium containing 10 µg/mL HRP (Sigma-Aldrich, St. Louis, MO, USA) was added into the upper chamber of transwell, and 2 mL of complete culture medium was added into the lower chamber. After incubated at 37°C for 1 h, 5 µL of culture medium from the lower chamber was collected and analyzed using tetramethylbenzidine (TMB) colorimetry method with a spectrophotometer at 370 nm. The final HRP flux (pmol/cm^2^/h) was calculated from the standard curve.

**Cell Transfection**

The short-hairpin RNAs (shRNAs) directed against RPL32P3 and HNF4G genes were ligated into GV102 vector (GeneChem, Shanghai, China) to construct the RPL32P3(-) and HNF4G(-) plasmid respectively. The shRNA directed against KMT2A gene was ligated into GV248 vector (GeneChem, Shanghai, China) to construct the KMT2A(-) plasmid. The shRNA directed against YBX2 gene was ligated into GV654 vector (GeneChem, Shanghai, China) to construct the YBX2(-) plasmid. The RPL32P3 gene full-length sequence, KMT2A and HNF4G gene coding sequence were ligated into GV658 vector (GeneChem, Shanghai, China) to construct the RPL32P3(+), KMT2A(+) and HNF4G(+) plasmid respectively. The plasmid with YBX2 gene coding sequence was ligated into GV417 vector (GeneChem, Shanghai, China) to construct the YBX2(+) plasmid. Empty vectors were used as their NCs.

ECs were seeded in 24-well plates and transfected using lipofectamine LTX (Life Technologies, Carlsbad, CA, USA) when the confluence reached 70-80%. The stably transfected cells were selected using G418 (0.4 mg/mL; Sigma-Aldrich, St. Louis, MO, USA) or puromycin (1 μg/mL; Sigma-Aldrich, St. Louis, MO, USA).

**Reporter Vector Construction and Dual Luciferase Reporter Assay**

The fragments of YBX2, ZO-1, occludin and claudin-5 promoters were amplified from human genomic DNA. Then the sequences were constructed into the pGL3 vector (Promega, Madison, WI, USA). Human full-length KMT2A and HNF4G were constructed into the pEX3 vector (Gene Pharma, Shanghai, China). HEK293T cells were seeded in 96-well plates. Both the dual luciferase vector and the plasmid vector were co-transfected into HEK293T cells using Lipofectamine 3000 (Life Technologies, Carlsbad, CA, USA). After 48 h, luciferase activity was analyzed by a dual-luciferase reporter assay system (Promega, Madison, WI, USA), which was recorded as the ratio of firefly luciferase activity to renilla luciferase activity.

**RIP Assay**

Magna RNA-binding protein immunoprecipitation kit (Millipore, Billerica, MA, USA) was applied to perform RIP assays according to the manufacturer’s protocol. Cell lysates were collected and incubated overnight with RIP buffer containing magnetic beads, which conjugated with KMT2A antibody (14197, Cell Signaling Technology, Danvers, MA, USA) or YBX2 antibody (13538-1-AP, Proteintech). IgG was used as the negative control. Samples were incubated with Proteinase K buffer and then immunoprecipitated RNA was isolated. Finally, the RNA concentration was purified and analyzed by qRT-PCR.

**RNA Pull-down Assay**

Pierce^TM^ Magnetic RNA-Protein Pull-Down Kit (Thermo Fisher Scientific, Waltham, MA, USA) was used to perform RNA pull-down assays according to the manufacturer’s protocol to validate the binding between RPL32P3 and KMT2A. In brief, the biotin-labelled RPL32P3 or anti-sense RNA were constructed and incubated with protein extract of GECs and magnetic beads at room temperature. Next, the beads-RNA-protein precipitates were obtained by low-speed centrifugation and purified by washing buffer. Finally, western blot assays were carried out to analyze the protein level after washing by elution buffer and GAPDH was used as the control. The binding between YBX2 and HNF4G mRNA was also investigated by the above method.

**Nascent RNA Capture**

Nascent RNAs were detected by Click-iT nascent RNA capture kit (Thermo Fisher Scientific, Waltham, MA, USA) following the manufacture’s protocols. We used 5-ethynyl uridine to label the nascent RNA, which was then isolated using streptavidin magnetic beads. Finally, Nascent RNAs were analyzed by qPCR.

**ChIP and ChIP-qPCR**

ChIP assay was performed using the SimpleChip Enzymatic Chromatin IP kit (Cell Signaling Technology, Danvers, MA, USA) according to the manufacturer’s protocol. Briefly, cells were crosslinked with 1% formaldehyde. The lysis buffer was used to lyse the cells, and then the micrococcal nuclease was used to digest the chromatin. Immunoprecipitation was incubated with KMT2A antibody (14197, Cell Signaling Technology), H3K4me3 antibody (9751, Cell Signaling Technology), HNF4G antibody (25801-1-AP, Proteintech) or normal rabbit IgG (negative control) followed by immunoprecipitation with Protein G Agarose Beads in each sample during an overnight incubation at 4℃ with gentle shaking. The 2% input reference was taken out and stored at -20℃ before adding antibody. The DNA crosslink was reversed by 5M NaCl and Proteinase K and the ChIP DNA was purified. Immunoprecipitated DNA was amplified by PCR using their specific primers. PCR product was visualized on a 3% or 2% agarose gel according to its size.

ChIP-qPCR analysis of DNA immunoprecipitated was used to quantify the relative DNA enrichment.

**ChIRP Assay**

ChIRP assay was conducted using the Magna ChIRP RNA Interactome Kit (Millipore, Billerica, MA, USA) according to the manufacturer’s protocols. ChIRP assay was undertaken with antisense probe sets against RPL32P3 or LacZ. Briefly, antisense DNA oligonucleotide probes specific for full-length RPL32P3 were designed on the following website: www.biosearchtech.com. And probes targeting LacZ were selected as nonspecific negative controls. Labelled probes according to their positions along the RNA. After crosslinking cells at room temperature, supplemented lysis buffer to lyse crosslinked cells to prepare cell lysate. Then sonicated crosslinked cell lysates to shear DNA. And hybridized biotinylated DNA probes to RNA and isolate bound chromatin. Finally, extracted the probes by streptavidin magnetic beads, and isolated the combined RNAs and DNAs for qRT-PCR. Data were normalized to the corresponding input control and expressed as a percentage of input.

**Analysis of Apoptosis by Flow Cytometry**

We stably transfected RPL32P3 knockdown, YBX2 knockdown and HNF4G knockdown, and co-transfected them in ECs. Then the BTB model in vitro was established by co-culture with U251 glioma cells, and the apoptosis of U251 cells was analyzed. After BTB models were established, 10 µmol/l of doxorubicin (DOX; Beyotime Institute of Biotechnology, Jiangsu, China) was added to the upper chamber of transwell. The apoptosis rates of U251 cells seeded in the lower chamber were detected 12 h later by using the Annexin V-PE/7AAD kit (Southern Biotech, Birmingham, AL, USA). The U251 cells in the lo­wer chamber were washed with PBS and centrifuged twice, then resuspended with Annexin V bounding buffer. Resuspended cells were stained with Annexin V-PE/7AAD for 15 min in the dark at room temperature according to the manufacturer’s instructions. Cell samples were analyzed by flow cytometry (BD Biosciences, San Jose, CA, USA) and apoptotic fractions were acquired.
