# Supplementary material for: Pseudogene RPL32P3 regulates the blood–tumor barrier permeability via the YBX2/HNF4G axis
Source: Cell Death Discov. 2021 Nov 24;7:367. doi: 10.1038/s41420-021-00758-9 (PMC8613260; doi:10.1038/s41420-021-00758-9)
Supplement: Supplementary file 2 — Supplemental Tables [file 41420_2021_758_MOESM2_ESM.docx]

Table S1. Primers used for qRT-PCR

| Gene | Sequence (5'→3') |
| --- | --- |
| RPL32P3 | F: GCCGAGAGGGAAAGGAGCATTC |
|  | R: TCCAGCACAGCCGACCTAGAG |
| RPL32 | F: GGCGGAAACCCAGAGGCATTG |
|  | R: GCTCCTTGACGTTGTGGACCAG |
| KMT2A | F: CTTCCAGATGGCCCCAAACC |
|  | R: CTTGCCTCGCTTCTTCCCAG |
| YBX2 | F: GGCGCAGAAGCCACTAATGT |
|  | R: GTTGCCCAGAGTCTTCAGCC |
| HNF4G | F: GGGTTTCTTCAGACGCAGCA |
|  | R: CCGAACTTCAGCTTGTGCCA |
| GAPDH | F: CGGATTTGGTCGTATTGGG |
|  | R: CTGGAAGATGGTGATGGGATT |
| ZO-1 | F: GGCGGATGGTGCTACAAGTGATG |
|  | R: AGGCTCAGAGGACCGTGTAATGG |
| occludin | F: TGGCTATGGCTACGGAGGCTATAC |
|  | R: CCAGAAGACTGAGCAGTTGGGTTC |
| claudin-5 | F: CGCCTTCCTGGACCACAACATC |
|  | R: AGAGCCAGCACCGAGTCGTAC |

Table S2. Sequences of shRNA template

| Gene |  | Sequence (5'→3') |
| --- | --- | --- |
| RPL32P3 | Sence | GATCCCACGGATGAATGGTCTGAGCATCTCGAGATGCTCAGACCATTCATCCGTTTTTTGGAT |
|  | Antisence | AGCTATCCAAAAAACGGATGAATGGTCTGAGCATCTCGAG ATGCTCAGACCATTCATCCGTGG |
| KMT2A | Sence | GATCCCGCCTCCATCAACAGAAAGGATCTCGAGATCCTTTCTGTTGATGGAGGCTTTTTGGAT |
|  | Antisence | AGCTATCCAAAAAGCCTCCATCAACAGAAAGGATCTCGAG ATCCTTTCTGTTGATGGAGGCGG |
| YBX2 | Sence | CCGGGCCCAGGTACCGAAGGCCTTTCTCGAGAAAGGCCTTCGGTACCTGGGTTTTTG |
|  | Antisence | AATTCAAAAAGCCCAGGTACCGAAGGCCTTTCTCGAGAAAGGCCTTCGGTACCTGGGC |
| HNF4G | Sence | GATCCCAAGCACCAGAAGAAGCACATTCTCGAGAATGTGCTTCTTCTGGTGCTTTTTTTGGAT |
|  | Antisence | AGCTATCCAAAAAAAGCACCAGAAGAAGCACATTCTCGAGAATGTGCTTCTTCTGGTGCTTGG |

Table S3. Primers used for ChIP assays

| Gene | Binding site or control | Sequence (5'→3') | Product  size (bp) | Annealing temperature (°C) |
| --- | --- | --- | --- | --- |
| YBX2 | PCR1 | F: GCGGTCACACTGTGAGGTTT | 201 | 55.6 |
| with KMT2A |  | R: ATCGGGCACAGTGGACAGAA |  |  |
|  | PCR2 | F: GACCGGCCCGTTTGATTTC | 219 | 54.5 |
|  |  | R: CCAATCCCAGCCCAGCAG |  |  |
| YBX2 | PCR1 | F: GGAGGCTGAGGAGGGAGAAT | 500 | 60.1 |
| with H3K4me3 |  | R: GGTTCTGGCAGTGAGTAGCA |  |  |
|  | PCR2 | F: GCAAATGGTCAGGATCATGTCA | 500 | 59.4 |
|  |  | R: CACAAGGTCTCCCTCTGTCG |  |  |
|  | PCR3 | F: TCAGCGAGGAAGCGGAATC | 500 | 60.1 |
|  |  | R: CACTCTTCGCCATCACACCA |  |  |
|  | PCR4 | F: TCCCGAACTCCTGACCTCAG | 500 | 60.0 |
|  |  | R: GTCCTCCGCGTCTCCTTC |  |  |
| ZO-1 | PCR1 | F: TCCTGTTCAGTTGCAGAGATGA | 152 | 56.4 |
|  |  | R: ACGCATGTCATCATAGCTCCT |  |  |
|  | PCR2 | F: AGGTCTTTCATCACCACCTTT | 247 | 55.8 |
|  |  | R: CTGGGCAACAAGAGCGAAAC |  |  |
|  | PCR3 | F: ATTCTTCTGCCTCAGCCTCC | 202 | 55.4 |
|  |  | R: TAAGAATTGTGGGCCAGGCG |  |  |
| occludin | PCR1 | F: AGACTCCACAGGCATGTTGG | 201 | 57.5 |
|  |  | R: TGTTGGTGGGAATGTGGAGC |  |  |
|  | PCR2 | F: TGGAAGCAGAAAAGTGTCCTGT | 101 | 55.7 |
|  |  | R: GAACGAGGTCCAGAGGGGA |  |  |
| claudin-5 | PCR1 | F: GCTGAGGTTGGATGAGCTGT | 139 | 57.4 |
|  |  | R: CTCACTGGTAACGAAGCCCC |  |  |
|  | PCR2 | F: ATGAGTGGCAGGAAAGGTGG | 232 | 56.8 |
|  |  | R: GCAGAGGAGTCTCAGGCTTC |  |  |

Table S4. Antisense DNA probes against RPL32P3 in ChIRP

| Probe number | Probe Sequence (5'→3') | Probe position |
| --- | --- | --- |
| 1 | CCGAGCAGGAAAAAGAAGCA | 41 |
| 2 | AGATGGCCACGGATTAAGAC | 375 |
| 3 | TGACATATCGGTCTGACTGG | 506 |
| 4 | AGGGCAGTCATTGAGGAATT | 663 |
| 5 | CTTGTTTGCTAGGTTCTGAC | 925 |
| 6 | TGTATAACACCTGCAGCTTG | 1029 |
| 7 | ACTGAGACCTCGAATGCTCC | 1129 |
| 8 | GACCTAGAGCTGGAAAGACC | 1229 |
| 9 | CTTCTGACCACTCTGAACAG | 1334 |
| 10 | CTAGATGGCAGAGAAGAGCT | 1436 |
| 11 | ACAATAGGGTAGGAGTTGCT | 1561 |
| 12 | TATCACCAAAACACTCCCTG | 2023 |

Table S5. Primers used for ChIRP

| Gene | Sequence (5'→3') |
| --- | --- |
| RPL32P3 | F: CCGAGAGGGAAAGGAGCATTC |
|  | R: GGAGCCCAGAAGCAGAGAA |
| YBX2 | F: TGGCTCATGGGAGCAAGT |
|  | R: GGGATCCAACCGCAGGATAG |
