# Supplementary figures and images for: Pseudogene RPL32P3 regulates the blood–tumor barrier permeability via the YBX2/HNF4G axis
Source: Cell Death Discov. 2021 Nov 24;7:367. doi: 10.1038/s41420-021-00758-9 (PMC8613260; doi:10.1038/s41420-021-00758-9)

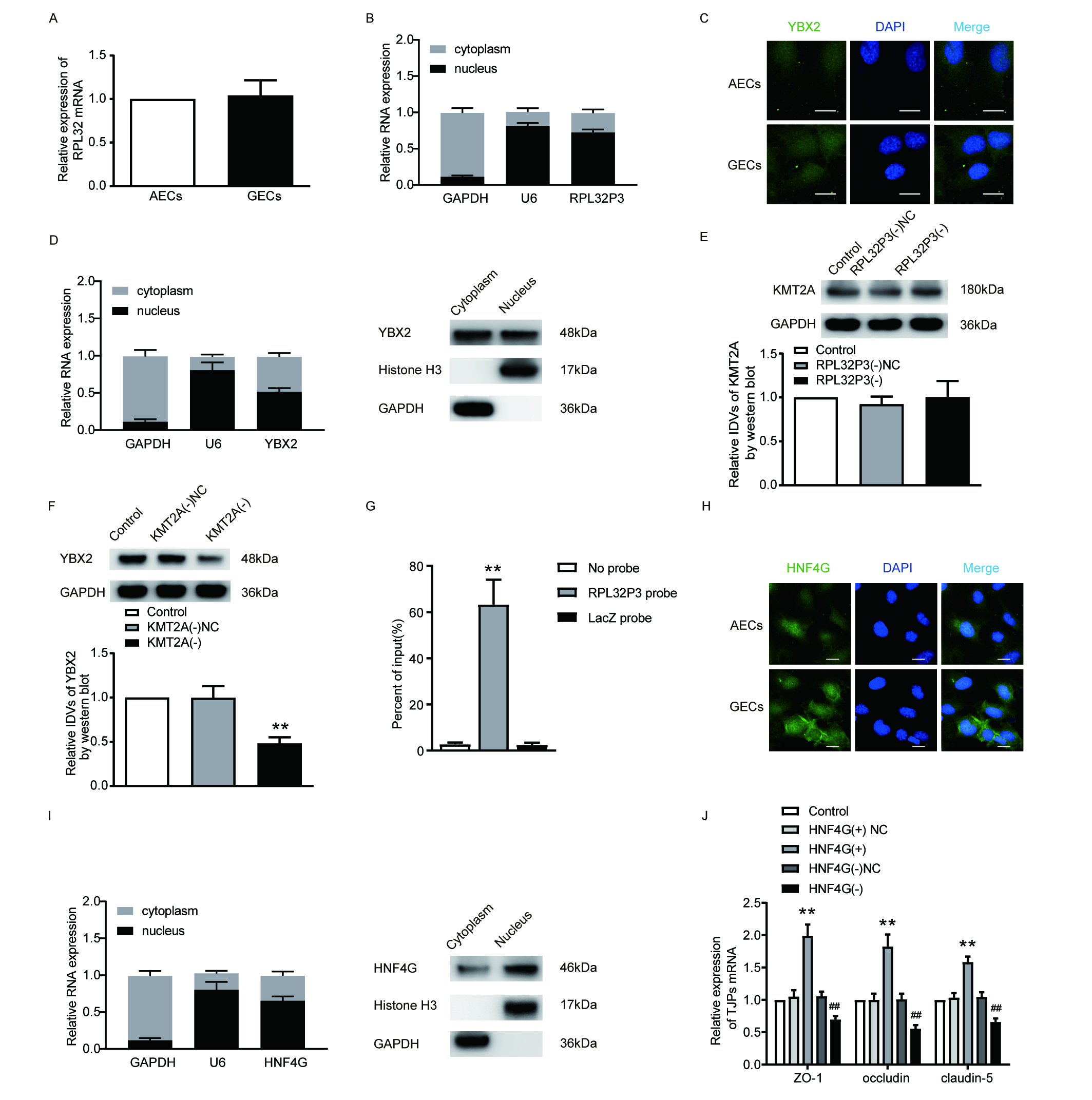

Supplement: Supplementary file 3 — Supplementary Figure S1 [file 41420_2021_758_MOESM3_ESM.tif]

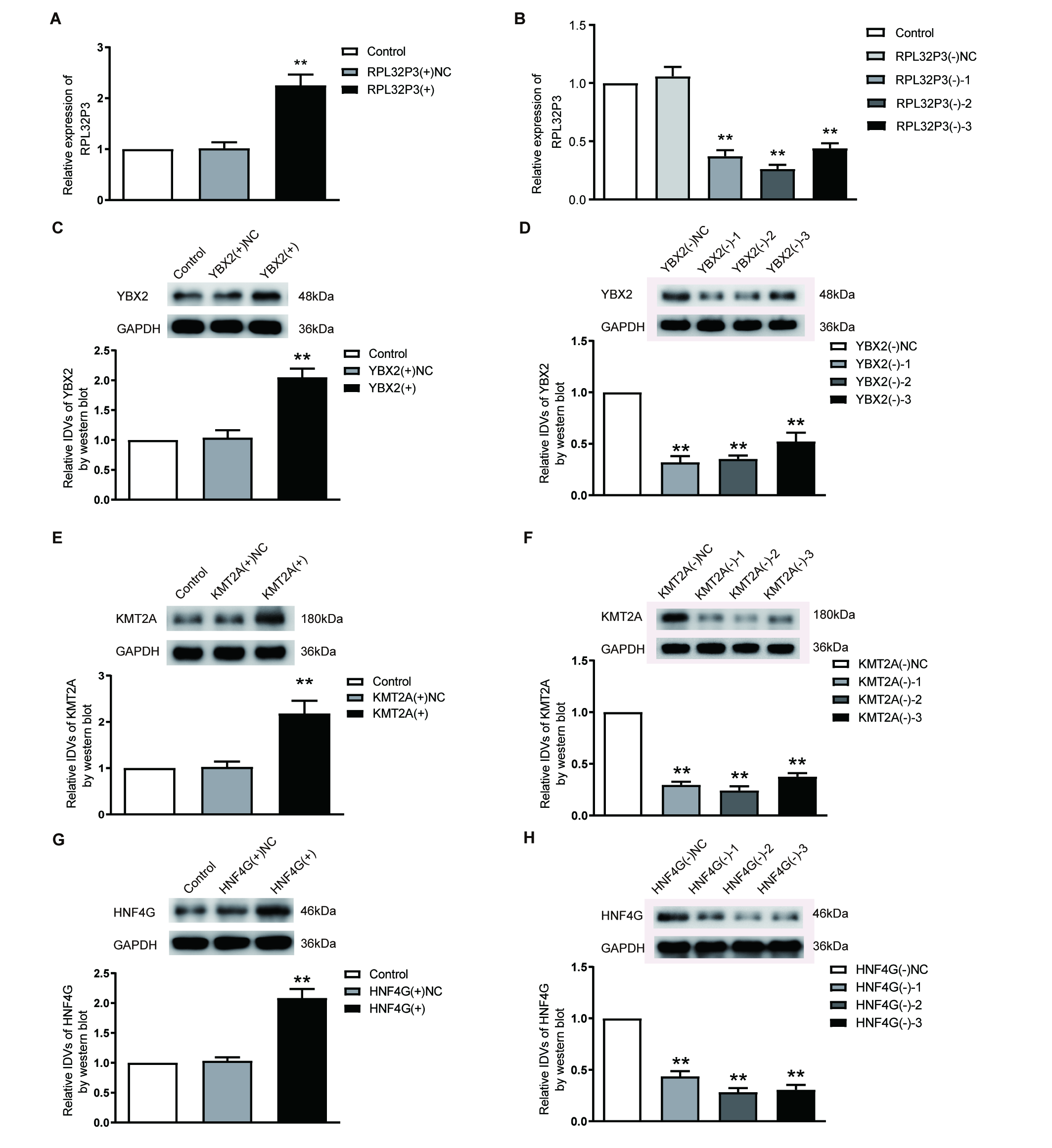

Supplement: Supplementary file 4 — Supplementary Figure S2 [file 41420_2021_758_MOESM4_ESM.tif]

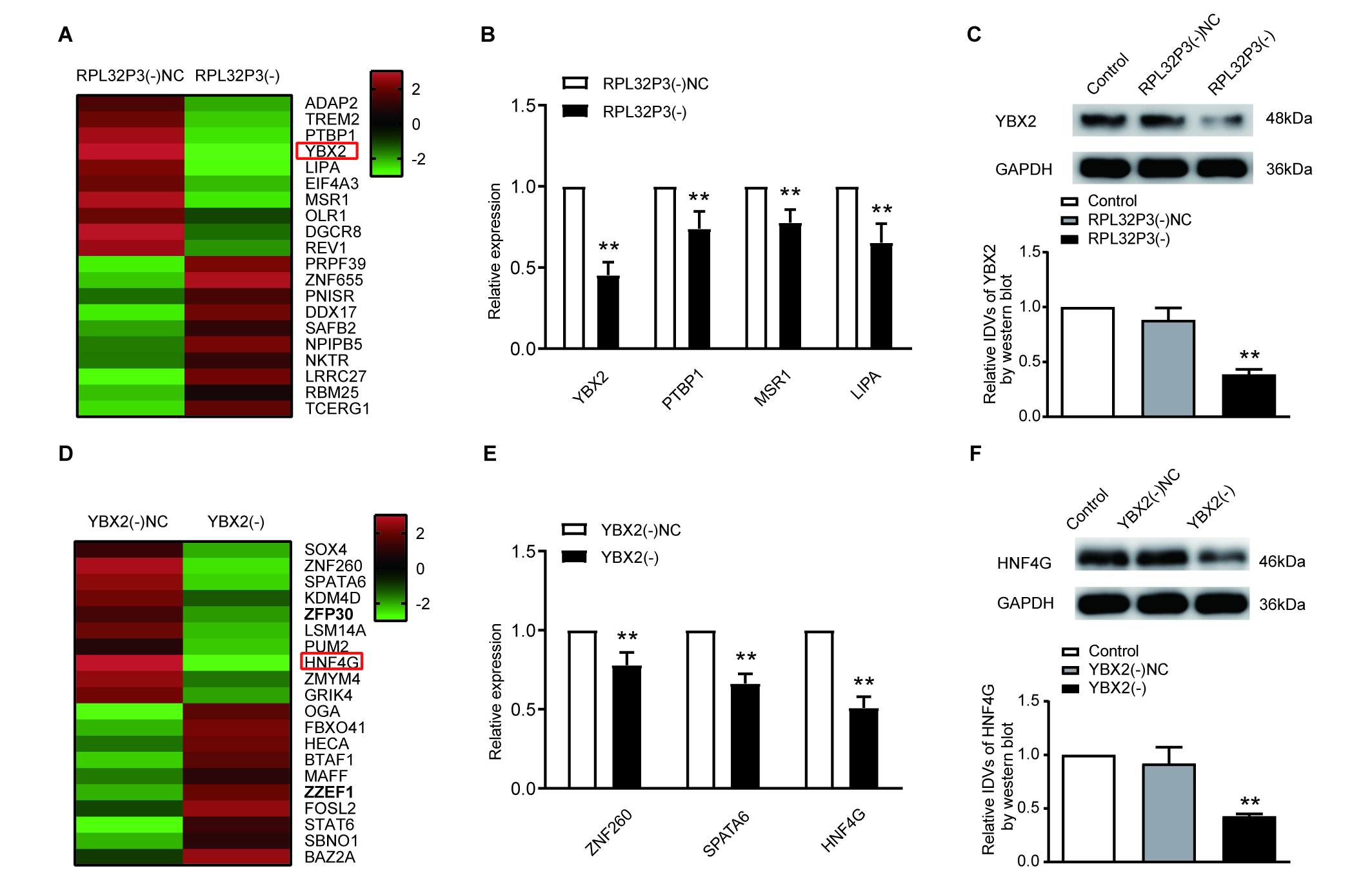

Supplement: Supplementary file 5 — Supplementary Figure S3 [file 41420_2021_758_MOESM5_ESM.tif]

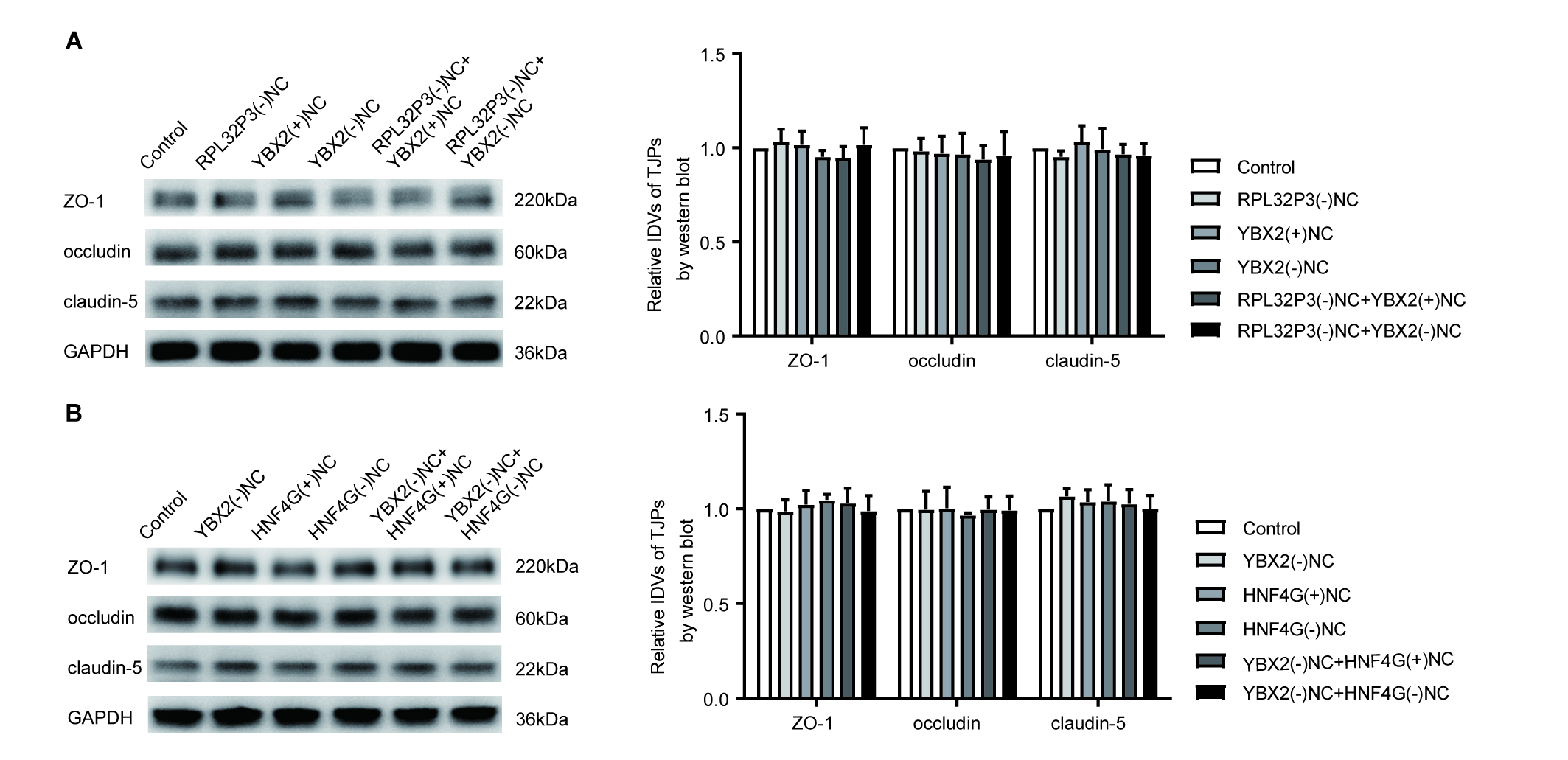

Supplement: Supplementary file 7 — Supplementary Figure S5 [file 41420_2021_758_MOESM7_ESM.tif]
