# Supplementary material for: Pseudogene RPL32P3 regulates the blood–tumor barrier permeability via the YBX2/HNF4G axis
Source: Cell Death Discov. 2021 Nov 24;7:367. doi: 10.1038/s41420-021-00758-9 (PMC8613260; doi:10.1038/s41420-021-00758-9)
Supplement: Supplementary file 8 — Supplementary Figure Legend [file 41420_2021_758_MOESM8_ESM.docx]

**Fig. S1**

(A) The mRNA expression level of RPL32 in AECs and GECs was measured by qRT-PCR. Data represent mean ± SD (n = 3, each). (B) Nucleus-cytoplasm separation assay was used to detect the subcellular location of RPL32P3 in GECs. Data represent mean ± SD (n = 3, each). (C) Immunofluorescence was performed to investigate the expression and subcellular location of YBX2 in AECs and GECs. Scale bar represents 30μm. (D) Nucleus-cytoplasm separation assay was used to detect the subcellular location of YBX2 in GECs. Data represent mean ± SD (n = 3, each). (E) Western blot was conducted to evaluate the protein expression of KMT2A after knockdown of RPL32P3. Data represent mean ± SD (n = 3, each). (F) Western blot was conducted to evaluate the protein expression of YBX2 after knockdown of KMT2A. Data represent mean ± SD (n = 3, each). **P<0.01 vs. KMT2A(-)NC group. (G) ChIRP assay showed that antisense probe against RPL32P3 could bind and capture RPL32P3 effectively. RNA was recovered with specific primers against RPL32P3 and then detected by qRT-PCR after reverse transcription. Data represent mean ± SD (n = 3, each group). **p < 0.01 vs. LacZ probe. (H) Immunofluorescence was performed to investigate the expression and subcellular location of HNF4G in AECs and GECs. Scale bar represents 30μm. (I) Nucleus-cytoplasm separation assay was performed to detect the subcellular location of RPL32P3 in GECs. Data represent mean ± SD (n = 3, each). (J) Effects of HNF4G knockdown on ZO-1, occludin, and claudin-5 mRNA expression levels determined by qRT-PCR. Data represent mean ± SD (n = 3, each group). **P < 0.01 vs. HNF4G(+)NC group, ^##^ P < 0.01 vs. HNF4G(-)NC group.

**Fig. S2 The transfection efficiencies of RPL32P3, KMT2A, YBX2 and HNF4G.**

(A) The transfection efficiency of RPL32P3 overexpression was detected by qRT-PCR. Data represent mean ± SD (n = 3, each group). **P < 0.01 vs. RPL32P3(+)NC group. (B) The transfection efficiency of RPL32P3 knockdown was detected by qRT-PCR. **P < 0.01 vs. RPL32P3(-)NC group. (C) The transfection efficiency of YBX2 overexpression was detected by western blot. Data represent mean ± SD (n = 3, each group). **P < 0.01 vs. YBX2(+)NC group. (D) The transfection efficiency of YBX2 knockdown was detected by western blot. Data represent mean ± SD (n = 3, each group). **P < 0.01 vs. YBX2(-)NC group. (E) The transfection efficiency of KMT2A overexpression was detected by western blot. Data represent mean ± SD (n = 3, each group). **P < 0.01 vs. KMT2A(+)NC group. (F) The transfection efficiency of KMT2A knockdown was detected by western blot. Data represent mean ± SD (n = 3, each group). **P < 0.01 vs. KMT2A(-)NC group. (G) The transfection efficiency of HNF4G overexpression was detected by western blot. Data represent mean ± SD (n = 3, each group). **P < 0.01 vs. HNF4G(+)NC group. (H) The transfection efficiency of HNF4G knockdown was detected by western blot. Data represent mean ± SD (n = 3, each group). **P < 0.01 vs. HNF4G(-)NC group.

**Fig. S3 Selection of YBX2 and HNF4G.**

(A) mRNA microarray analysis data in GECs after downregulation of RPL32P3. Red indicates high expression and green indicates low expression. (B) qRT-PCR was conducted to validate the selected molecules (n=3, each group). **P<0.01 vs. RPL32P3(-)NC group. (C) Effect of RPL32P3 knockdown on the protein expression of YBX2 via western blot. Data represent mean ± SD (n = 3, each). **P<0.01 vs. RPL32P3(-)NC group. (D) mRNA microarray analysis data in GECs after downregulation of YBX2. Red indicates high expression and green indicates low expression. (E) qRT-PCR was conducted to validate the selected molecules (n=3, each group). **P<0.01 vs. YBX2(-)NC group. (F) Effect of YBX2 knockdown on the protein expression of HNF4G determined by western blot. Data represent mean ± SD (n = 3, each). **P<0.01 vs. YBX2(-)NC group.

**Fig. S4 Bioinformatics database and software prediction results.**

(A) The predicted H3K4me3 modification sites on the YBX2 promoter according to bioinformatics tool ENCODE project. (B) The interaction between KMT2A and YBX2 promoter were predicted via Animal TFDB database. (C-E) The predicted binding sites with HNF4G on the TJPs promoter according to JASPAR database. (F) The CpG islands on the YBX2 promoter were predicted by bioinformatics software MethPrimer.

**Fig. S5 Effects of control groups on TJPs determined by western blot.**

(A) Effects of the control groups of RPL32P3 and YBX2 on the expression levels of ZO-1, occludin and claudin-5 determined by western blot. Data represent mean ± SD (n = 3, each). (B) Effects of the control groups of YBX2 and HNF4G on the expression levels of ZO-1, occludin and claudin-5 determined by western blot. Data represent mean ± SD (n = 3, each).

**List of Abbreviations**

Blood-tumor barrier (BTB), blood-brain barrier (BBB), endothelial cells (ECs), glioma-exposed endothelial cells (GECs), astrocyte-exposed ECs (AECs), tight junction-related proteins (TJPs), lysine methyltransferase 2A (KMT2A), Y-box binding protein 2 (YBX2), hepatocyte nuclear factor 4 gamma (HNF4G), doxorubicin (DOX), noncoding RNAs (ncRNAs), long ncRNAs (lncRNAs), ribosomal protein L32 (RPL32), tri-methylation of lysine 4 on histone H3 (H3K4me3), transcription start site (TSS), human embryonic kidney 293T (HEK293T), fluorescence in situ hybridization (FISH), integrated light density values (IDVs), transendothelial electric resistance (TEER), horseradish peroxidase (HRP), short-hairpin RNAs (shRNAs), RNA immunoprecipitation (RIP), chromatin immunoprecipitation (ChIP), chromatin isolation by RNA purification (ChIRP), coding sequences (CDS).
