# Supplementary material for: Pseudogene RPL32P3 regulates the blood–tumor barrier permeability via the YBX2/HNF4G axis
Source: Cell Death Discov. 2021 Nov 24;7:367. doi: 10.1038/s41420-021-00758-9 (PMC8613260; doi:10.1038/s41420-021-00758-9)
Supplement: Supplementary file 9 — cddiscovery-author-contribution-form [file 41420_2021_758_MOESM9_ESM.pdf]

DECLARATION OF CONTRIBUTIONS TO ARTICLE

ADMC

Manuscript Number:	Journal Name:	
CDDISCOVERY-21-1975	*Cell Death Discovery*	(the 'Journal')

Proposed Title of the Contribution:

Pseudogene RPL32P3 regulates the blood-tumor barrier permeability via the YBX2/HNF4G axis (the 'Contribution)

Author(s):

Ye Ding, Xiaobai Liu, Chunqing Yang, Xuelei Ruan, Di Wang, Yunhui Liu, Xiuli Shang, Qianshuo Liu,Shuyuan Shen, Lu Zhu, Yixue Xue (the 'Authors')

For all *CDDis*covery articles, each person named as an author in the published version must be able to show he or she has contributed substantially to the article.

Authorship credit should be based on 1) substantial contributions to conception and design, acquisition of data, or analysis and interpretation of data; 2) drafting the article or revising it critically for important intellectual content; and 3) final approval of the version to be published. Authors should meet conditions 1, 2 and 3.

Any person who cannot be shown to have made a substantial contribution to the article cannot be listed as an author in the final version. The name of any person who is deemed to have made a minor contribution can, however, appear in the Acknowledgments section of the article.

Please complete the table below to indicate the contributions of all named authors to the manuscript.

Author Full Name:	Specification of Contribution to the Manuscript:
Ye Ding	acquisition of data; analysis and interpretation of data; drafting of the manuscript
Xiaobai Liu	acquisition of data; drafting of the manuscript
Chunqing Yang	acquisition of data
Xuelei Ruan	acquisition of data
Di Wang	acquisition of data
Yunhui Liu	critical revision of the manuscript for important intellectual content; administrative, technical, and material suppor
Xiuli Shang	analysis and interpretation of data
Qianshuo Liu	analysis and interpretation of data
Shuyuan Shen	analysis and interpretation of data
Lu Zhu	analysis and interpretation of data
Yixue Xue	conception and design; critical revision of the manuscript for important intellectual content; administrative, technical, and material support; final approval of the version to be published

Please complete the table below to indicate the contributions of all named authors to the figures.

Figure 1:

Y.D., X.L., C.Y., X.R., D.W., Q.L., L.Z., and X.S. generated the data, Y.D. assembled the figure.

Figure 2:

Y.D., X.L., C.Y., X.R., D.W., Q.L., L.Z., and X.S. generated the data, Y.D. assembled the figure.

Figure 3:

X.L., C.Y., and S.S. generated the data, Y.D. assembled the figure.

Figure 4:

Y.D., X.L., C.Y., X.R., D.W., and X.S. generated the data, Y.D. assembled the figure.

Figure 5:

Y.D., X.L., C.Y., X.R., D.W., and X.S. generated the data, Y.D. assembled the figure.

Figure 6:

Y.D., X.L., C.Y., X.R., D.W., and X.S. generated the data, Y.D. assembled the figure.

Signed for and on behalf of the Author(s):	Print Name:	Date:
	Xue Yixue	2021.10.25
